# Study on the Microstructure of Mg-4Zn-4Sn-1Mn-xAl As-Cast Alloys

**DOI:** 10.3390/ma16216979

**Published:** 2023-10-31

**Authors:** Junlin Liu, Zhiwei Du, Yonggang Peng, Rongguang Jia, Xiaolei Han, Minglong Ma, Ting Li

**Affiliations:** 1 China GRINM Group Co., Ltd., National Center of Analysis and Testing for Non-Ferrous Metals & Electronic Materials, Beijing 100088, China; liujunlin@cutc.net (J.L.); pengyonggang@cutc.net (Y.P.); hanxiaolei@gbtcgroup.com (X.H.); maminglong@grinm.com (M.M.); liting@gbtcgroup.com (T.L.); 2 Guobiao (Beijing) Testing & Certification Co., Ltd., Beijing 110407, China; 3 General Research Institute for Nonferrous Metals, Beijing 100088, China; 4 China United Test & Certification Co., Ltd., Beijing 110407, China; 5 State Key Laboratory of Non-Ferrous Metals & Processes, GRIMAT Engineering Institute Co., Ltd., Beijing 101407, China

**Keywords:** Mg-Zn-Sn-Mn-Al alloy, microstructure, second phases, micro-alloying

## Abstract

In this study, the microstructure of the Mg-4Zn-4Sn-1Mn-xAl (x = 0, 0.3 wt.%, denoted as ZTM441 and ZTM441-0.3Al) as-cast alloys was investigated using scanning electron microscopy (SEM), focused-ion/electron-beam (FIB) micromachining, transmission electron microscopy (TEM), and high-angle annular dark-field scanning transmission electron microscopy (HAADF-STEM). The analysis results revealed that the microstructure of the ZTM441 and ZTM441-0.3Al as-cast alloys both mainly consist of the α-Mg matrix, skeleton-shaped MgZn_2_ eutectic texture, block-shaped Mg_2_Sn, and Zn/Sn-rich nanoscale precipitate bands along the grain boundary and the interdendrite. Nanoscale α-Mn dispersoids formed in the grain in the ZTM441 alloy, while no α-Mn formed in the ZTM441-0.3Al alloy instead of nanoscale Al_3_Mn_2_ particles. In the ZTM441 as-cast alloy, part of the Zn element is dissolved into the α-Mn phase, and part of the Mn element is dissolved into the MgZn_2_ phase, but in the ZTM441-0.3Al alloy, there are no such characteristics of mutual solubility. Zn and Mn elements are easy to combine in ZTM441 as-cast alloy, while Al and Mn are easy to combine in ZTM441-0.3Al as-cast alloy. The Mg-Zn phases have not only MgZn_2_-type crystal structure but also Mg_4_Zn_7_- and Mg_149_Zn-type crystal structure in the ZTM441-0.3Al as-cast alloy. The addition of Al changes the combination of Mn and Zn, promotes the formation of Al_3_Mn_2_, and the growth of the grain.

## 1. Introduction

Magnesium (Mg) alloys have been widely used in transportation, aerospace, and electronics industries because of their low density, high specific strength, good damping performance, and suitability for surface treatments [1,2,3,4,5]. However, the adoption of magnesium alloys remains limited for low absolute strength at room temperature and elevated temperature. Although Mg-RE (rare earth) alloys have better comprehensive properties such as high strength and heat resistance [6], the RE elements are heavy, expensive, and scarce. It is necessary to select free-RE elements to substitute RE elements to decrease the cost and the weight of high strength, heat-resistant magnesium alloys.

Mg-Sn-based alloys are promising candidates and have received considerable attention in recent years [7]. Mg-Sn-based alloys are aging strengthening alloys, and the maximum equilibrium solid solubility of the Sn element in the α-Mg matrix is approximately 14.48 wt.% [8]. Mg-Sn-based alloys mainly include Mg-Sn-Zn, Mg-Sn-Al, Mg-Sn-Ca alloys, and so on [9,10,11,12]. Among them, Mg-Sn-Zn alloys demonstrate better high strength and heat-resistant properties [13,14,15]. In order to further increase the strength of the Mg-Sn-Zn alloys at elevated temperatures, researchers have focused on the Zn/Sn mass ratio and micro-alloying in Mg-Sn-Zn alloys. Zn/Sn mass ratio can change the type of the Zn-rich and Sn-rich phases while micro-alloying elements can form new second phases [16,17,18,19,20,21,22,23,24,25,26,27]. Mg-Zn-Sn alloys with the Zn/Sn mass ratio of 1:1, and Mg-Sn-Zn-Mn alloys have excellent strength at elevated temperatures [28,29,30]. In Mg alloys containing Zn or Sn elements, such as Mg-Zn-Sn, Mg-Zn, and Mg-Sn alloys, the second phases usually include MgZn_2_, Mg_4_Zn_7_, Mg_2_Zn_11_, Mg_2_Sn, etc. [31,32,33,34,35,36,37,38]. In Mg alloys, the Mn element usually forms the α-Mn particles, promotes grain refinement, and improves the corrosion resistance [39,40]. In addition, the Al element is a commonly used micro-alloying element in Mg alloys and forms Mg_17_Al_12_ Al-Mn phases, etc. [41,42,43,44,45].

Studies on the microstructure of Mg-6Zn-4Sn-1Mn-xAl (wt.%) alloys have been reported [46,47,48]. These studies focused on the second phase and properties of the extruded and aged states of the Mg-6Zn-4Sn-1Mn-xAl series alloys after homogenization heat treatment. The results show that the grain sizes decrease and then increase with the increase in Al element content, and the addition of Al elements can promote the formation of second phases such as Al_8_Mn_5_, Al_11_Mn_4_, Mg_32_(Al,Zn)_49_, Al_2_Mg_5_Zn_2_, and MgZn. However, in this study, the Al-Mn phases possess an Al_3_Mn_2_ structure, which is not consistent with that reported in the literature [46,47,48,49]. And the combination of Mn and Zn would be changed for the Al addition. Therefore, it can be deduced that the formation of the second phase is sensitive to the contents of the alloying elements. It is necessary to investigate the second phase in the Mg-4Zn-4Sn-1Mn-xAl (x = 0, 0.3 wt.%) alloys.

In the present study, the microstructure of the Mg-4Zn-4Sn-1Mn-xAl (x = 0, 0.3 wt.%) as-cast alloys was investigated using Powder X-ray diffraction (PXRD), scanning electron microscopy (SEM), focused-ion beam (FIB), and transmission electron microscopy (TEM) techniques. The distribution, composition, and crystal structure of the complex second phase in the as-cast alloy were systematically analyzed in the two alloys. The effect of the addition of Al element on the formation law of the second phases in the Mg-4Zn-4Sn-xAl (x = 0, 0.3 wt.%) as-cast alloys was explored. This study will provide a basis for the design of subsequent heat treatment and hot working processes.

## 2. Materials and Methods

The nominal chemical compositions of experimental alloys were Mg-4Zn-4Sn-1Mn (wt.%, denoted as ZTM441) and Mg-4Zn-4Sn-1Mn-0.3Al (wt.%, denoted as ZTM441-0.3Al). The raw chemicals for the alloy were supplied by Trillion Metals Co., Ltd. (Beijing, China). The alloys were prepared by melting pure Mg (≥99.99 wt.%), pure Zn (≥99.99 wt.%), pure Sn (≥99.99 wt.%), pure Al (≥99.99 wt.%), and Mg-3% wt.% Mn in a medium-frequency electromagnetic induction furnace under the protection of Ar and C_2_H_2_F_2_ atmosphere with a ratio of 1:20, and the size of the ingots was φ 90 × 150 mm^3^. The actual chemical compositions of the alloy were measured using Agilent 5110 ICP-AES (Agilent, Santa Clara, CA, USA) and are listed in Table 1.

The phases of the alloys were analyzed with the SmartLab X-ray diffraction (XRD) using Cu-Ka radiation (λ = 1.5406 Å) with a scanning angle from 20°–90°. The backscattered electron (BSE) images and electron backscatter diffractometer (EBSD) results were obtained using JSM-7900F SEM at 20 kV, which was provided by JEOL (Tokyo, Japan) and equipped with the X-Max 80 energy dispersive spectrometer (EDS) and Symmetry electron backscatter diffractometer. Bright-field (BF) images, high-angle annular dark-field scanning transmission electron microscopy (HAADF-STEM) images, high-resolution transmission electron microscopy (HRTEM) images, and the selected area electron diffraction (SAED) patterns were obtained using Talos F200X TEM equipped with Super X four-detector energy spectrometer (Thermo Fisher Scientific, Waltham, MA, USA) at 200 kV.

SEM samples were prepared via mechanical polishing with grinding and polishing machines. Labopol-30 and Tagramin-30 EBSD samples were electrochemically polished, followed by mechanical polishing, in a solution AC2 cooled at −30 °C, using a voltage of 20 V and a current of 0.5 mA for 40 s. TEM samples were mechanically ground down to 50 μm and then cut into 3 mm diameter thin foils, and then prepared via twin-jet electropolishing (MTP-1A Double-Sprayer, JIAODA, Jiading, Shanghai, China) in an electrolyte containing 85 vol.% C_2_H_5_OH, 5 vol.% HClO_4_, and 10 vol.% C_6_H_14_O_2_ at −30 °C and the subsequent ion thinning (695.0 PIPS II ion thinning apparatus, Gatan, Pleasanton, CA, USA) at −140 °C with a voltage of 2 KeV. TEM samples for the gray contrast band and site-specific intergranular second phase observation were prepared using a focused-ion/electron dual beam system (Helios 5 CX, Thermo Fisher Scientific, Waltham, MA, USA).

## 3. Results

### 3.1. XRD Results

**Figure 1:**
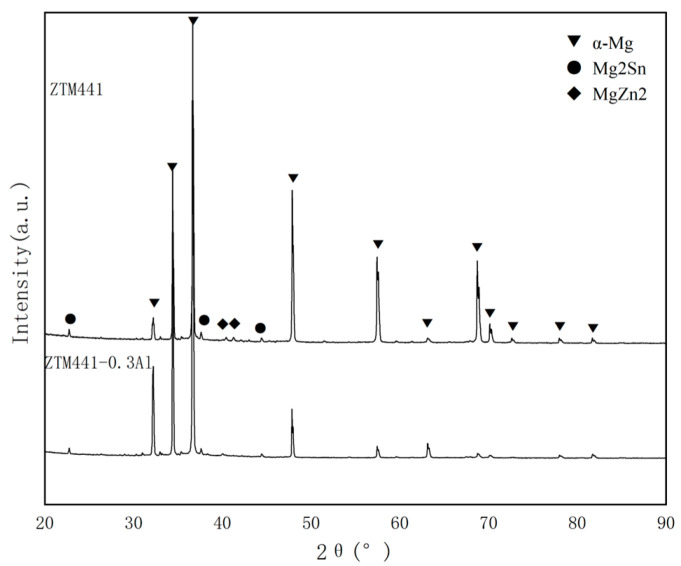
XRD patterns of ZTM441-xAl (x = 0, 0.3 wt.%) as-cast alloys.

Figure 1 shows the XRD patterns of the two as-cast alloys. The XRD results were identified by the data from the Inorganic Crystal Structure Database (ICSD) with reference numbers 170902 for α-Mg, 642855 for Mg_2_Sn, and 46006 for MgZn_2_, respectively. The analyses revealed that ZTM441 and ZTM441-0.3Al as-cast alloys mainly consist of α-Mg matrix, Mg_2_Sn, and MgZn_2_. No Mg_17_Al_12_ phases appeared in the ZTM441-0.3Al as-cast alloy, which can be attributed to the low Al/Zn ratio [16,50].

### 3.2. SEM Results

**Figure 2:**
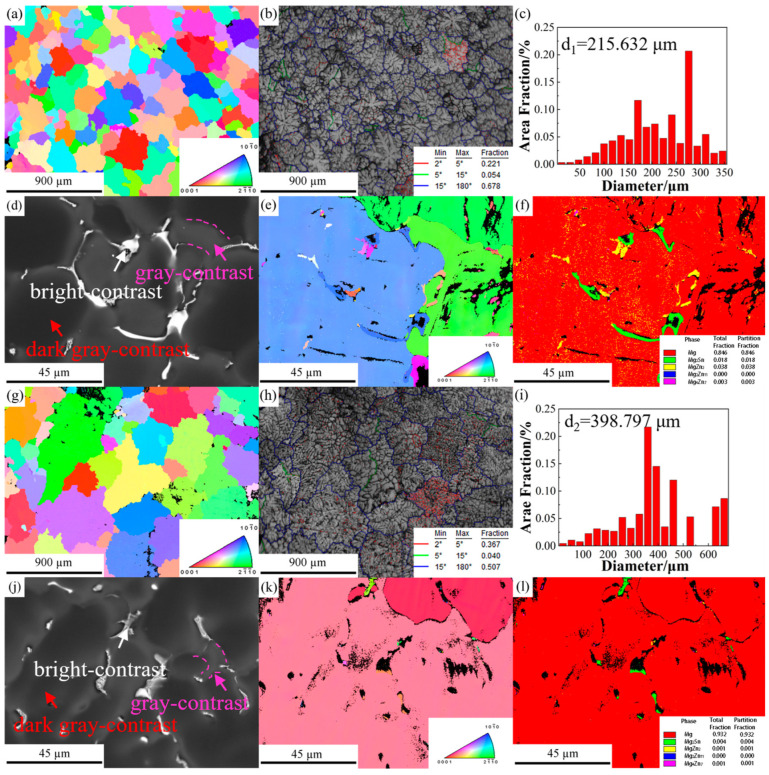
EBSD maps of ZTM441 and ZTM441-0.3Al alloys. (**a**–**f**) ZTM441, (**g**–**l**) ZTM441-0.3Al; (**a**,**g**) IPF maps, (**b**,**h**) distribution of GBs with IQ, (**c**,**i**) grain size distribution histogram, (**d**,**j**) BSE image at high magnification, (**e**,**k**) IPF maps at high magnification, and (**f**,**l**) second phase distribution map at high magnification.

The EBSD results were analyzed using the OIM 8 software. Figure 2a–c,g–i show the inverse pole figure (IPF) maps, image quality (IQ) maps with grain boundaries (GBs), and grain size distribution histograms of ZTM441 and ZTM441-0.3Al as-cast alloys. The misorientation angles between the adjacent grains are used to identify the low-angle grain boundaries (LAGBs, 2° ≤ θ ≤ 15°) and high-angle grain boundaries (HAGBs, θ > 15°). The average grain size weighted by area (denoted as the average grain size) was calculated by the formula which is as follows:

(1) $$\bar{d}=\frac{\sum_{i=1}^{N}A_{i}d_{i}}{\sum_{i=1}^{N}A_{i}}$$

where *A_i_* is the area of grain *i* and *d_i_* is the equivalent diameter of grain *i*. The equivalent diameter of a particular grain is calculated by determining the area of a grain and then assuming the grain is a circle. The average grain size is 215.632 μm (d_1_), the fraction of HAGBs is 67.8%, and the fraction of LAGBs is 27.5% in the ZTM441 alloy, while the average grain size is 398.797 μm (d_2_), the fraction of HAGBs is 50.7%, and the fraction of LAGBs is 40.7% in ZM441-0.3Al alloy. Adding Al makes the grain grow, and the fraction of HAGBs decreases. There are a large number of dendritic crystals formed during the solidification processes in ZTM441 and ZTM441-0.3Al as-cast alloys, as shown in Figure 2b,h. Figure 2d,j show BSE images of the two alloys, and corresponding IPF maps and phase distribution maps are shown in Figure 2e,f and Figure 2k,l, respectively. The results indicate that the second phases are distributed at the grain boundary and the interdendrite in the two alloys, and the second phases are mainly Mg_2_Sn and MgZn_2_ in ZTM441 and Mg_2_Sn in ZTM441-0.3Al.

**Figure 3:**
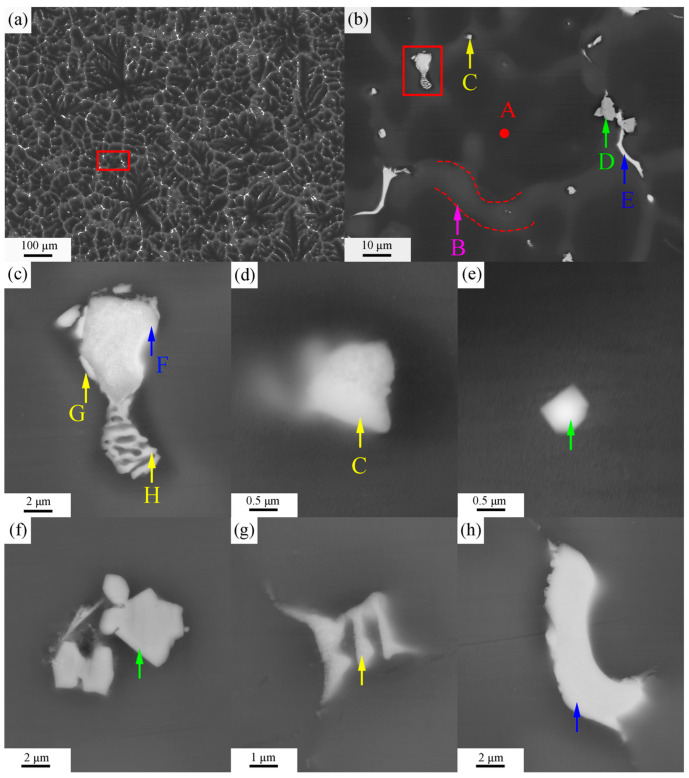
BSE images of ZTM441 as-cast alloy. (**a**) Low magnification BSE image, (**b**) the high magnification image of the red area in (**a**), (**c**) BSE image of the co-distributed block-shaped Sn-rich and skeletal Zn-rich phases of the red area in (**b**), and the BSE images of separately distributed (**d**) Zn-rich particles at position C in (**b**), (**g**) skeletal Zn-rich phase, (**e**) Mn-rich particle, (**f**) angular Mn-rich phase, and (**h**) block-shaped Sn-rich phase.

**Figure 4:**
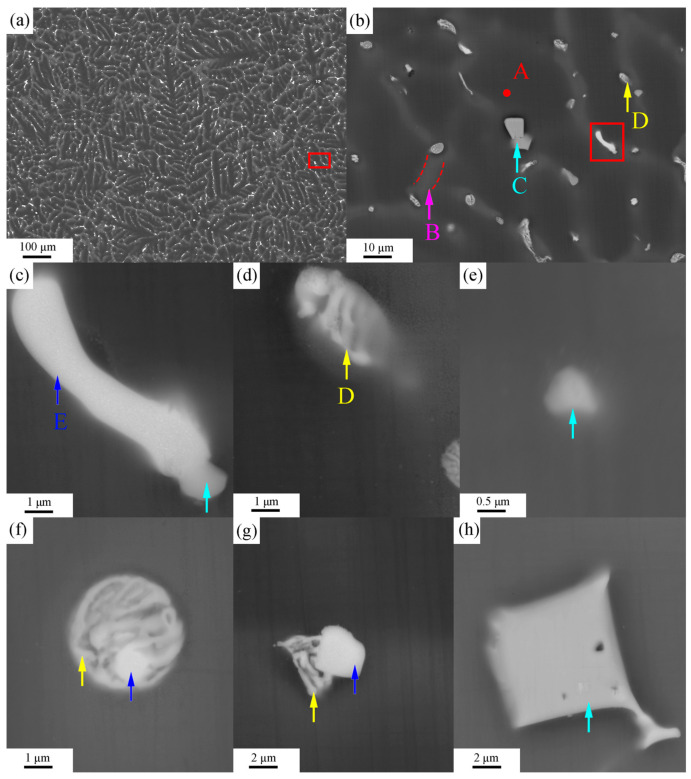
BSE images of ZTM44-0.3Al as-cast alloy, as follows: (**a**) low magnification BSE image, (**b**) the high magnification image of the red area in (**a**), and the BSE images of (**c**) the co-distributed block-shaped Sn-rich and Al-Mn-rich phases in the red area in (**b**), (**f**,**g**) the co-distributed block-shaped Sn-rich and skeletal Zn-rich phases, and the BSE images of separately distributed, (**d**) skeletal Zn-rich phase, (**e**) Al-Mn-rich particles, and the (**h**) angular Al-Mn-rich phase.

Figure 3 and Figure 4 show BSE images of the ZTM441 and ZTM441-0.3Al as-cast alloys, respectively. And the corresponding EDS analysis results of the two alloys are shown in Table 2 and Table 3, respectively. The microstructure of the two alloys mainly consists of three contrasts (Figure 3a and Figure 4a): dark gray-contrast, gray-contrast, and bright-contrast. According to BSE images and EDS results, it can be seen that (1) the phase with dark gray contrast is an α-Mg matrix in the two alloys; and (2) the phases with bright-contrast are skeletal Zn-rich phase (marked by yellow arrows in Figure 3), block-like Sn-rich phase (marked by blue arrows in Figure 3), and angular Mn-rich phase (marked by green arrows in Figure 3) in the ZTM441 alloy, while they are skeletal Zn-rich eutectic phases (marked by yellow arrows in Figure 4); block-like Sn-rich phases (marked by blue arrows in Figure 4); and angular Al-Mn-rich phases (marked by azure arrows in Figure 4) in the ZTM441-0.3Al alloy.

**Figure 5:**
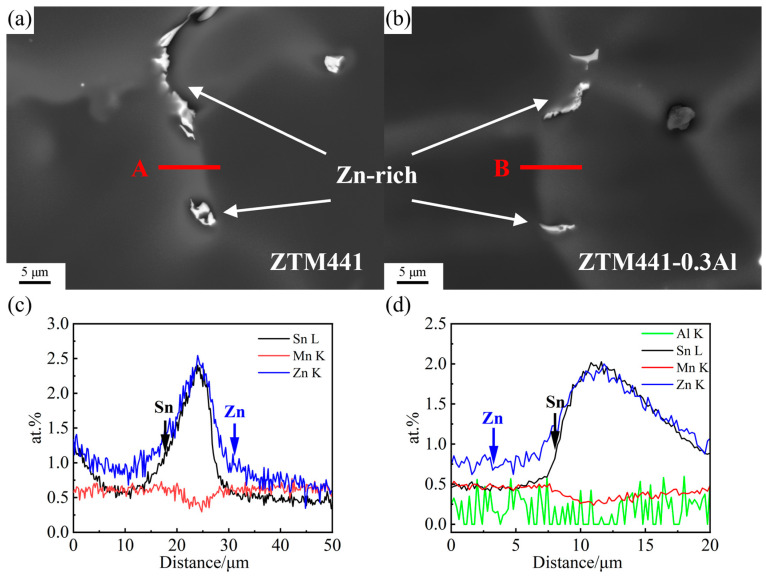
(**a**,**b**) BSE images of ZTM441 and ZTM441-0.3Al as-cast alloys; (**c**) EDS line scanning results of Zn, Sn, and Mn profile along the red line in the gray area (B region in Figure 3b) of ZTM441alloy and (**d**) the EDS line scanning results of Zn, Sn, Mn, and Al profile along the red line in the gray area (B region in Figure 4b) of ZTM441-0.3Al alloy.

The high magnification BSE images of the gray-contrast bands in ZTM441 and ZTM441-0.3Al as-cast alloys are shown in Figure 5a,b, respectively. The EDS line scanning results of the Zn, Sn, Mn, and Al profile along the red line (labeled A in Figure 5a and B in Figure 5b) in the gray-contrast bands are shown in Figure 5c,d. It shows that the gray contrast bands are enriched with Zn and Sn elements. The gray-contrast band similar to zone labeled B in ZTM441-0.3Al was sampled by FIB as the object, and its composition and structure were analyzed via TEM in Section 3.3 below.

### 3.3. TEM Results

#### 3.3.1. ZTM441

**Figure 6:**
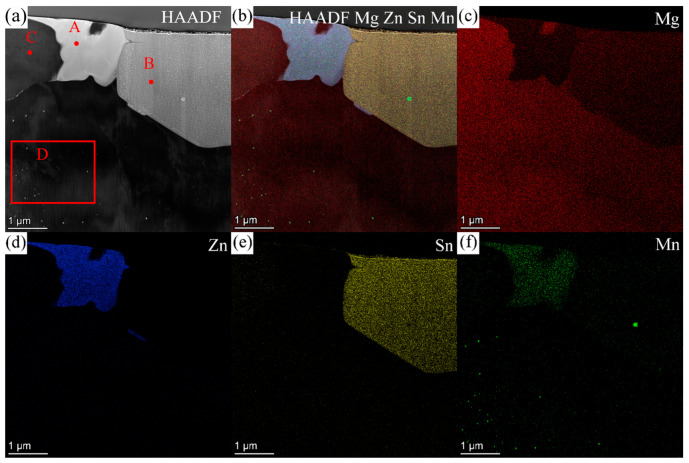
(**a**) HAADF image of the second phase in ZTM441 as-cast alloy, (**b**) mixed map of HAADF image and the EDS maps of (**c**) Mg, (**d**) Zn, (**e**) Sn, and (**f**) Mn.

Figure 6 shows the HAADF image and EDS maps for the second phases in ZTM441 as-cast alloy, and Table 4 shows the corresponding composition of the labeled second phases in Figure 6a. The Zn, Sn, and Mn contents (at.%) in the α-Mg matrix at position C in Figure 6a are 3.02, 1.26, and 0.31, respectively. The contents (at.%) of Mg, Zn, Sn, and Mn for position A are 27.98, 66.24, 0.05, and 5.73, and for position B are 68.94, 0.20, 30.33, and 0.53, which are close to the composition of MgZn_2_ and Mg_2_Sn phase, respectively. The content of Mn in MgZn_2_ was detected to be 5.73 at.%, which is lower than the maximum solubility of Mn in MgZn_2_, 14.0 at.% [51]. Meanwhile, nanoscale Mn-rich particles occurred in the grain, which is beneficial to refining grain [52].

**Figure 7:**
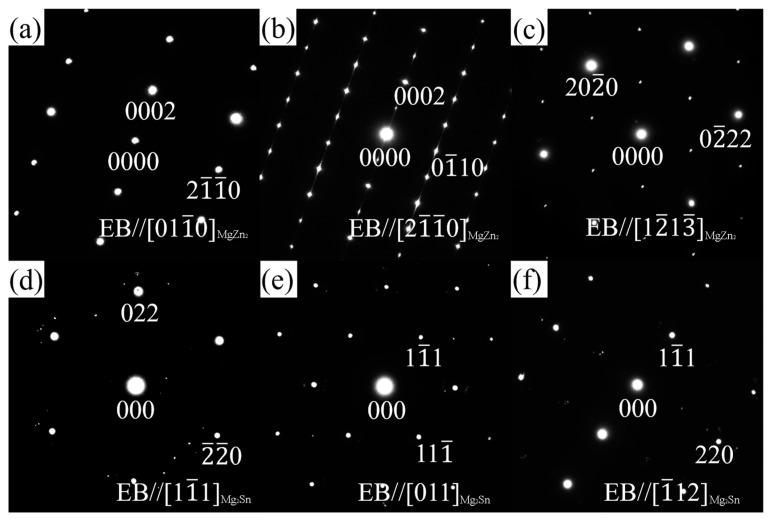
SAED patterns of MgZn_2_ (region A in Figure 6a) along (**a**) [01$\overset{\_}{1}$0], (**b**) [2$\overset{\_}{1}$$\overset{\_}{1}$0], and (**c**) [1$\overset{\_}{2}$1$\overset{\_}{3}$] zone axis and Mg_2_Sn (region B in Figure 6a) along (**d**) [1$\overset{\_}{1}$1], (**e**) [011], and (**f**) [$\overset{\_}{1}$12] zone axis in ZTM441 as-cast alloy.

**Figure 8:**
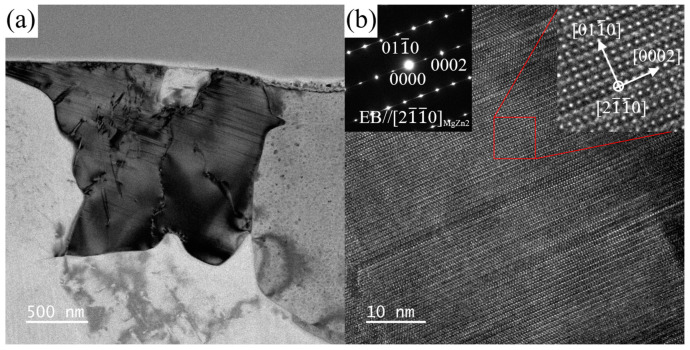
(**a**) BF image and (**b**) HRTEM image of MgZn_2_ phase along [2$\overset{\_}{1}$$\overset{\_}{1}$0]${}_{{MgZn}_{2}}$ in ZTM441 as-cast alloy.

The SAED patterns of the Zn-rich phase and Sn-rich phase shown in Figure 6a along three different zone axes were acquired via a series of tilting (Figure 7). Combining the EDS results and the magnitude of the reciprocal vector and the angle between the vectors, Figure 7a–c were indexed as the SAED patterns along [01$\overset{\_}{1}$0]${}_{{MgZn}_{2}}$, [2$\overset{\_}{1}$$\overset{\_}{1}$0]${}_{{MgZn}_{2}}$, and [1$\overset{\_}{2}$1$\overset{\_}{3}$]${}_{{MgZn}_{2}}$ for the Zn-rich phase, and Figure 7d,e along [1$\overset{\_}{1}$1]${}_{{Mg}_{2}Sn}$, [011]${}_{{Mg}_{2}Sn}$, and [$\overset{\_}{1}$12]${}_{{Mg}_{2}Sn}$ for the Sn-rich phase. The Zn-rich phase at position A in Figure 6a almost conforms to the crystal structure of MgZn_2_ phase, which has a close-packed hexagonal structure with a space group of P63/mmc. The calculated lattice parameters are a = b = 5.985 Å and c = 9.284 Å, slightly different from the theoretical value (a = b = 5.223 Å and c = 8.566 Å [53]), which may be caused by the solid solution of Mn element in the MgZn_2_ phase and the measurement error of the reciprocal vector. The Sn-rich phase at position B in Figure 6a almost conforms to the crystal structure of the Mg_2_Sn phase, which has a face-centered cubic (FCC) structure with a space group of Fm $\overset{\_}{3}$ m. The calculated lattice parameters are a = b = c = 7.381 Å, slightly different from the theoretical value (a = b = c = 6.759 Å [54]). Figure 8 shows the BF image, SAED pattern, and the HRTEM image of the MgZn_2_ phase along the [2$\overset{\_}{1}$$\overset{\_}{1}$0]${}_{{MgZn}_{2}}$ zone axis. Stacking faults can be observed in the MgZn_2_ particle.

**Figure 9:**
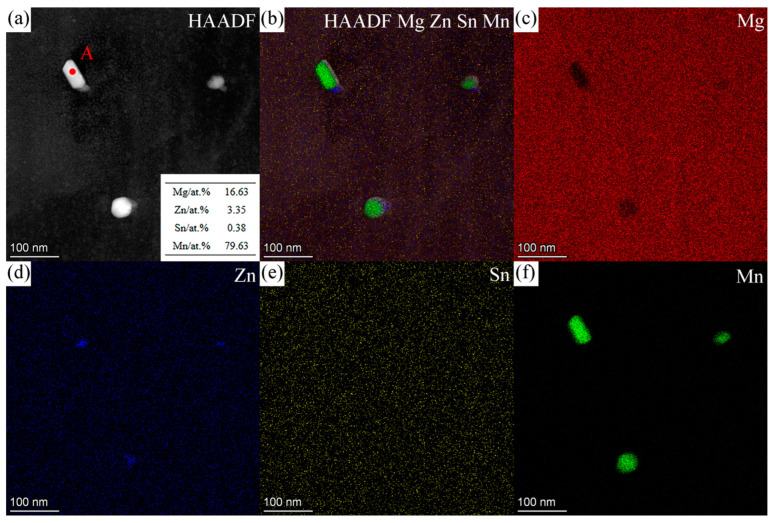
(**a**) HAADF image and the EDS quantitative results of nanoscale Mn-rich phase at position A in ZTM441 as-cast alloy, and the (**b**) mixed map of HAADF image and the EDS maps of (**c**) Mg, (**d**) Zn, (**e**) Sn, and (**f**) Mn.

**Figure 10:**
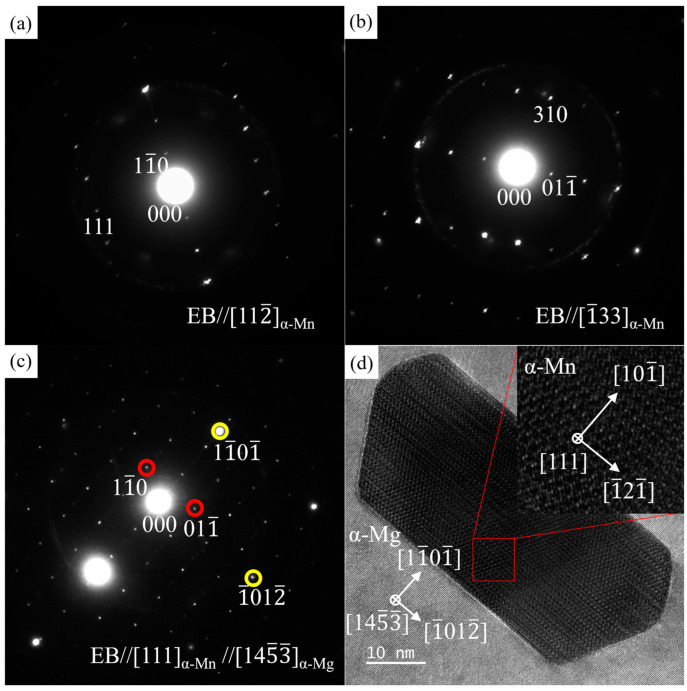
SAED patterns and HRTEM image of the nanoscale Mn-rich phase in the α-Mg matrix for ZTM441 as-cast alloy. (**a**) along [11$\overset{\_}{2}$]_α-Mn_, (**b**) along [$\overset{\_}{1}$33]_α-Mn_, (**c**) superposition SAED pattern along [111]_α-Mn_, (**d**) HRTEM image along [111]_α-Mn_.

Figure 9 shows the HAADF image and corresponding EDS results for nanoscale Mn-rich particles in the grain, which is similar to the phases in the red box marked as D in Figure 6a. The contents (at.%) of Mg, Zn, Sn, and Mn at position A in Figure 9a are 16.63, 3.35, 0.38 and 79.63, close to the α-Mn phase. Figure 10a–c shows the SAED patterns along [11$\overset{\_}{2}$]${}_{\alpha –Mn}$, [$\overset{\_}{1}$33]${}_{\alpha –Mn}$, and [111]${}_{\alpha –Mn}$ of the Mn-rich phase at position A (Figure 9a) acquired via a series of tilting. The Mn-rich phase almost conforms to the crystal structure of the α-Mn phase, which has a body-centered cubic (BCC) crystal structure with a space group of I $\overset{\_}{4}$3m. The calculated lattice parameters are a = b = c = 10.108 Å, slightly different from the theoretical value (a = b = c = 8.913 Å [55]), which may be caused by the solid solution of Zn element in the α-Mn phase. In Figure 10c, not only is the SAED pattern of α-Mn shown but also the high-index zone axis SAED pattern of the α-Mg matrix along [14$\overset{\_}{5}$$\overset{\_}{3}$]${}_{\alpha –Mg}$ is superimposed. Figure 10d shows the corresponding HRTEM image, which indicates that the orientation relationship between the α-Mg matrix and Mn-rich phase is [111]${}_{\alpha –Mn}$//[14$\overset{\_}{5}$$\overset{\_}{3}$]${}_{\alpha –Mg}$, (10$\overset{\_}{1}$)${}_{\alpha –Mn}$//(1$\overset{\_}{1}$0$\overset{\_}{1}$)${}_{\alpha –Mg}$.

#### 3.3.2. ZTM441-0.3Al

**Figure 11:**
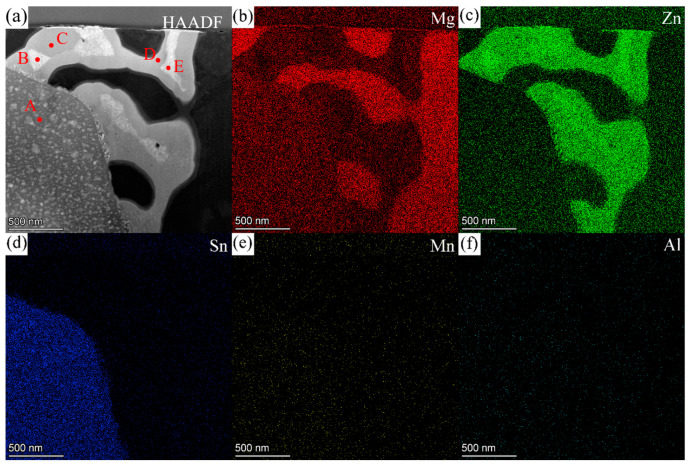
(**a**) HAADF image of the second phase in ZTM441-0.3Al as-cast alloy and the EDS maps of (**b**) Mg, (**c**) Zn, (**d**) Sn, (**e**) Mn, and (**f**) Al.

Figure 11 shows the HAADF image and EDS maps for the second phases in ZTM441-0.3Al as-cast alloy, and Table 5 shows the corresponding composition of the labeled second phases in Figure 11a. According to EDS quantitative results, the contents (at.%) of Mg, Zn, Sn, Mn, and Al at position A in Figure 11a are 59.49, 0.25, 39.97, 0.11, and 0.18, close to Mg_2_Sn phase. The contents (at.%) at position B are 20.84, 78.81, 0.07, 0.16, and 0.12; at position C are 37.69, 61.39, 0.05, 0.08, and 0.79; at position D are 29.34, 69.52, 0.08, 0.12, and 0.80; and at position E are 35.75, 63.52, 0.07, 0.07, and 0.59. Mn is almost non-existent in the Zn-rich phase due to the Mn mainly forming the Al-Mn-rich phase in ZTM441-0.3Al as-cast alloy, which is different from that in the ZTM441 as-cast alloy.

**Figure 12:**
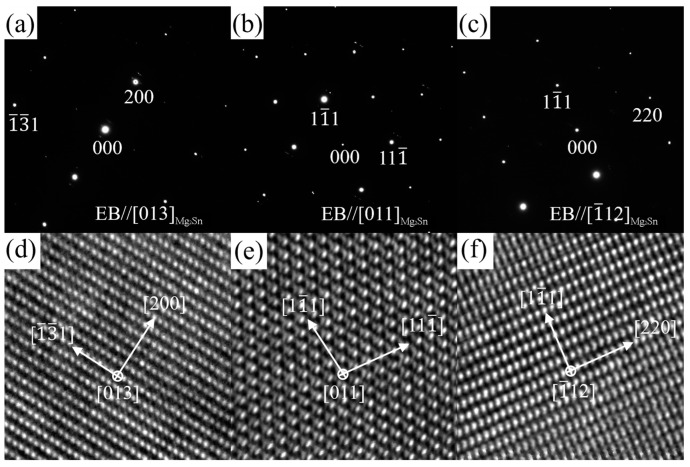
SAED patterns and HRTEM images of Mg_2_Sn phase (region A in Figure 10a) in ZTM441-0.3Al as-cast alloy taken along (**a**,**d**) [013]${}_{{Mg}_{2}Sn}$, (**b**,**e**) [011]${}_{{Mg}_{2}Sn}$, and (**c**,**f**) [$\overset{\_}{1}$12]${}_{{Mg}_{2}Sn}$ zone axis.

In order to determine the crystal structures of the phases at positions A, B, C, D, and E in Figure 11a, the SAED patterns along different zone axes were obtained via a series of tilting. Combining the EDS results, the SAED patterns shown in Figure 12a–c were, respectively, indexed as along [013]${}_{{Mg}_{2}Sn}$, [011]${}_{{Mg}_{2}Sn}$, and [$\overset{\_}{1}$12]${}_{{Mg}_{2}Sn}$ for the Sn-rich phase at position A. The crystal structure of the Sn-rich phase almost conforms to Mg_2_Sn phase, which has an FCC structure with a space group of Fm $\overset{\_}{3}$ m. The calculated lattice parameters are a = b = c = 7.175 Å, close to those in ZTM441 as-cast alloy (a = b = c = 7.381 Å) in Figure 6, slightly different from the theoretical value (a = b = c = 6.759 Å). Figure 12d–f shows the HRTEM images of the Mg_2_Sn phase taken along the [011]${}_{{Mg}_{2}Sn}$, [013]${}_{{Mg}_{2}Sn}$, and [$\overset{\_}{1}$12]${}_{{Mg}_{2}Sn}$ zone axis, which further indicate that the Sn-rich phase mainly has an Mg_2_Sn-type structure, and certain regions were damaged due to electron beam irradiation during FIB sample preparation or TEM observation (see Figure 11a).

**Figure 13:**
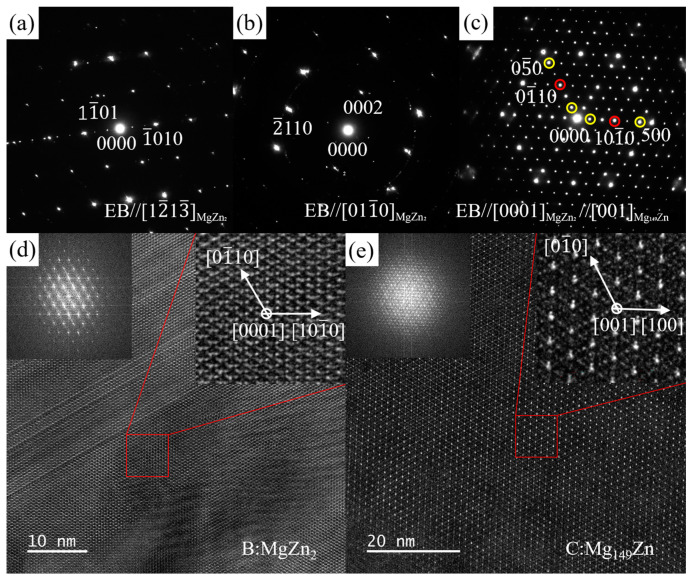
SAED patterns of Zn-rich phase (regions B and C in Figure 11a) along (**a**) [1$\overset{\_}{2}$1$\overset{\_}{3}$]${}_{{MgZn}_{2}}$, (**b**) [01$\overset{\_}{1}$0]${}_{{MgZn}_{2}}$ and the (**c**) superposition SAED pattern along [0001]${}_{{MgZn}_{2}}$ zone axis, and the HRTEM images and corresponding FFT electron diffraction patterns along [0001]${}_{{MgZn}_{2}}$ zone axis at position (**d**) B and (**e**) C in the ZTM441-0.3Al as-cast alloy.

Figure 13a,b shows the SAED patterns corresponding to position B along the [1$\overset{\_}{2}$1$\overset{\_}{3}$]${}_{{MgZn}_{2}}$ and [01$\overset{\_}{1}$0]${}_{{MgZn}_{2}}$ zone axis. Figure 13c shows the superposition SAED pattern of positions B and C along the [0001]${}_{{MgZn}_{2}}$ zone axis. Figure 13d,e show the HRTEM images and corresponding fast Fourier transform (FFT) electron diffraction patterns along the [0001]${}_{{MgZn}_{2}}$ zone axis of positions B and C, respectively (see Figure 11a). Obviously, regions B and C belong to two types of second phase. It was further confirmed that the diffraction spots marked with the red and yellow circles in Figure 13c were contributed by positions B and C, respectively. The Zn-rich phase at position B has the same crystal structure as the MgZn_2_ phase, and the calculated lattice parameters are a = b = 5.367 Å, c = 9.466 Å. The HRTEM images show the MgZn_2_ phase in ZTM441-0.3Al as-cast alloy contains fewer defects compared with it in ZTM441 as-cast alloy. The crystal structure of the second phase at position C almost conforms to the Mg_149_Zn phase, which is an α-Sm derived structure. The Mg_149_Zn phase has a triclinic crystal structure with a space group of P1, and the calculated lattice parameters are a = b = 14.525 Å, c = 14.933 Å, slightly different from the theoretical value (a = b = 15.96 Å, c = 15.63 Å, α = β = 90°, γ = 120° [56]). Studies on the Mg_149_Zn structure in magnesium alloys have rarely been reported.

**Figure 14:**
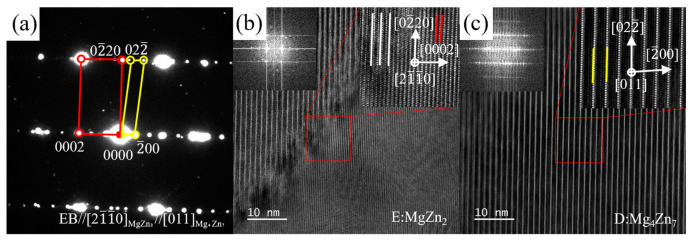
(**a**) The superposition SAED pattern of Zn-rich phase (regions D and E in Figure 11a) taken along [011]${}_{{Mg}_{4}{Zn}_{7}}$ and [2$\overset{\_}{1}$$\overset{\_}{1}$0]${}_{{MgZn}_{2}}$ zone axis, and the HRTEM images and corresponding FFT electron diffraction patterns of (**b**) MgZn_2_ phase (position E in Figure 11a) and (**c**) Mg_4_Zn_7_ (position D in Figure 11a) along [011]${}_{{Mg}_{4}{Zn}_{7}}$ and [2$\overset{\_}{1}$$\overset{\_}{1}$0]${}_{{MgZn}_{2}}$ zone axis.

Figure 14 shows the SAED pattern, HRTEM images, and FFT electron diffraction patterns of the area containing position D and E along the [2$\overset{\_}{1}$$\overset{\_}{1}$0]${}_{{MgZn}_{2}}$//[011]${}_{{Mg}_{4}{Zn}_{7}}$ zone axis. Based on the FFT electron diffraction patterns (inset in Figure 14b,c), it was confirmed that the diffraction spots in Figure 14a marked with red and yellow circles are contributed by positions E and D, respectively. The Zn-rich phase at position D almost conforms to the crystal structure of the Mg_4_Zn_7_ phase, which has a monoclinic structure with a space group of C2/m. The calculated lattice parameters are a = 25.820 Å, slightly different from the theoretical value (a = 25.96 Å [57,58]). The Zn-rich phase at position E also has the MgZn_2_ structure as that at position B. The coherent interfaces along [011]${}_{{Mg}_{4}{Zn}_{7}}$, and [2$\overset{\_}{1}$$\overset{\_}{1}$0]${}_{{MgZn}_{2}}$ are shown in Figure 14b. Mg_4_Zn_7_ and MgZn_2_ usually co-exist because of their similar formation energy [59,60]. MgZn_2_ and Mg_4_Zn_7_ phases usually precipitate after heat treatment or deformation for certain magnesium alloys containing Zn [58,61,62,63]; however, these phases have rarely been reported in the as-cast alloy.

**Figure 15:**
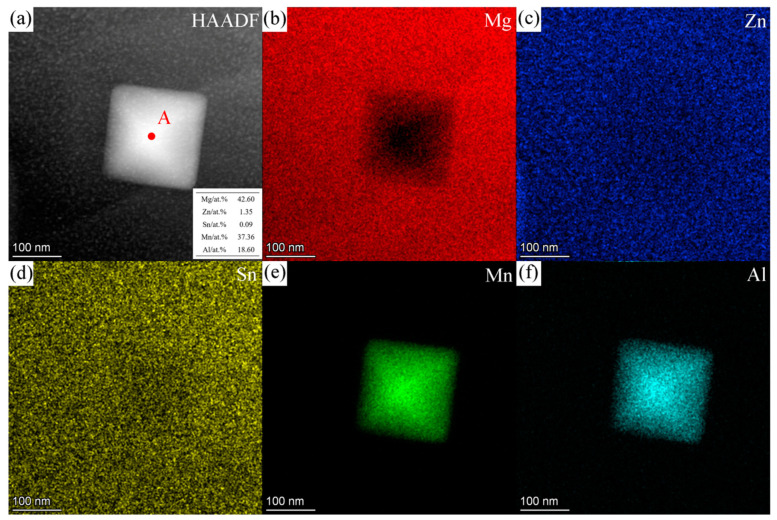
(**a**) HAADF image and the EDS quantitative results (inset) of the Al-Mn-rich phase in ZTM441-0.3Al cast alloy at position A and the EDS maps of (**b**) Mg, (**c**) Zn, (**d**) Sn, (**e**) Mn, and (**f**) Al.

**Figure 16:**
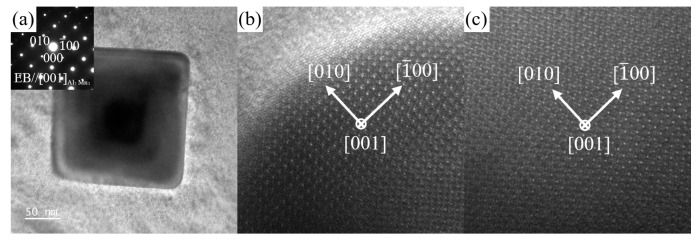
(**a**) BF image and the SAED pattern of the Al-Mn-rich phase in the ZTM441-0.3Al alloy taken along [001]${}_{{Al}_{3}{Mn}_{2}}$ zone axis in region A in Figure 15a and the HRTEM images (**b**) at the edge of the phase along [001]${}_{{Al}_{3}{Mn}_{2}}$ zone axis and (**c**) in the phase along [001]${}_{{Al}_{3}{Mn}_{2}}$ zone axis.

Figure 15 shows the HAADF image and corresponding EDS results for the square-shaped Al-Mn-rich phase. The contents (at.%) of Mg, Zn, Sn, Mn, and Al are 42.60, 1.35, 0.09, 37.36, and 18.60 at position A in Figure 15a, with the atomic ratios Mn: Al = 2:1 and Mn: (Al, Mg) = 2:3. Figure 16 shows the SAED pattern, BF image, and HRTEM images of the square-shaped Al-Mn-rich phase taken along the [001]${}_{{Al}_{3}{Mn}_{2}}$ zone axis. It was determined that the Al-Mn-rich phase almost conforms to the crystal structure of Al_3_Mn_2_, which has a cubic structure with a space group of P4-13$\overset{\_}{2}$. The calculated lattice parameters are a = b = c = 6.6287 Å, slightly different from the theoretical value (a = b = c = 6.3951 Å [64]). The Al-Mn phases are dominated by Al_8_Mn_5_ and Al_11_Mn_4_ in the magnesium alloys [42,65,66,67]. The formation of Al_3_Mn_2_ in magnesium alloys has rarely been reported.

**Figure 17:**
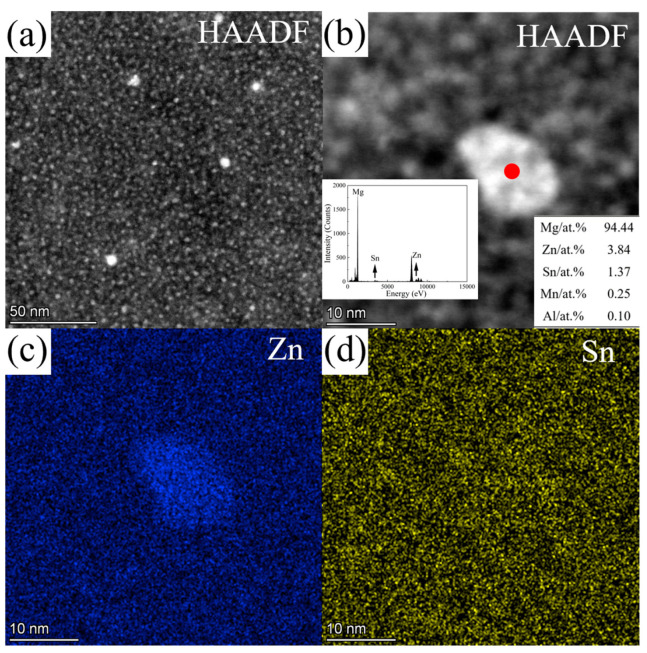
(**a**,**b**) HAADF images and corresponding EDS maps of (**c**) Zn element and (**d**) Sn element for the phases in gray contrast in ZTM441-0.3Al as-cast alloy.

The TEM samples containing the gray contrast bands were obtained via FIB site-specific lifting in the ZTM441-0.3Al as-cast alloy. Figure 17 shows the HAADF images and the corresponding EDS results for the Zn and Sn elements in the nanoscale phase in the gray contrast bands. The EDS quantitative results are similar to those of the α-Mg matrix (see Figure 6a) due to the beam extension. The corresponding EDS maps of Zn and Sn elements show that the gray contrast band mainly consists of the Zn-rich phase, contradicting those of the EDS line scanning results in Figure 5, which show Zn and Sn elements are rich in the gray contrast bands. It is possible that the Sn element escapes due to electron/ion beam irradiation during FIB sample preparation or TEM observation. Therefore, it is considered that the gray contrast may consist of both Zn-rich and Sn-rich phases or Zn-Sn-rich phases.

## 4. Discussion

In this study, the microstructure of Mg-4Zn-4Sn-1Mn-xAl (x = 0, 0.3 wt.%) as-cast alloys was comparatively analyzed using a combination of SEM, EBSD, FIB, and TEM techniques. The intergranular second phases and the gray-contrast zones were especially fixed-point lifted out by FIB. Then, the types of the second phases were determined using SAED, HRTEM, HAADF-STEM, and EDS. The results show that the intergranular second phases in the ZTM441 as-cast alloy are skeleton-shaped α-Mg+MgZn_2_ eutectic texture and block-shaped Mg_2_Sn and are skeleton-shaped α-Mg+Mg-Zn eutectic texture consisted of MgZn_2_, Mg_4_Zn_7_, and Mg_149_Zn, and block-shaped Mg_2_Sn in the ZTM441-0.3Al alloy. Among them, the Mg_149_Zn was not reported. Therefore, the distribution and crystal structures of the intergranular second phases are complex. The gray-contrast zones along the grain boundary and the interdendrite are composed of the nanoscale Zn/Sn-rich precipitates. Otherwise, the dispersoids, α-Mn and Al_3_Mn_2_, formed in the grain in the ZTM441 and ZTM441-0.3Al alloys, respectively.

With the addition of the Al element, firstly, the grain sizes increase from 215.632 μm in the ZTM441 to 398.797 μm in the ZTM441-0.3Al. It is considered that the addition of Al element inhibits the formation of α-Mn and weakens the refining grain effect of dispersoid. Although Al_3_Mn_2_ may also play the role of α-Mn in refining grains, via extensive observation, the size of Al_3_Mn_2_ is larger than that of α-Mn, and the number density is lower than that of α-Mn. Secondly, it can be found that the α-Mn dispersoids disappear and are replaced by Al_3_Mn_2_ particles. Ye, Hou, and Deng reported the Al_8_Mn_5_ and Al_11_Mn_4_ in the Mg-6Zn-4Sn-1Mn-xAl alloys [46,47,48,49]. However, no studies have been reported on the formation of the Al_3_Mn_2_ phase in Mg-Zn-Sn-Mn-Al alloy. Therefore, the structure of the Al-Mn phase is very sensitive to the content of alloying elements. In addition, in ZTM441 alloy without Al, the Mg-Zn phase contains a certain amount of Mn (5.73 at.%), and a certain amount of Zn (3.35 at.%) is dissolved in α-Mn at the same time. However, in ZTM441-0.3Al alloy, the Mg-Zn phase nearly does not contain Mn, and the Mn element will combine with Al to form the Al-Mn phase. Therefore, it is believed that Al changes the combination of alloying elements.

In this study, the lattice constants of the second phase were determined using the SAED patterns. When excluding the measurement error of the SAED patterns, the lattice constants of some of the second phases still deviate significantly from the theoretical values, which are mainly attributed to the solid solution of other alloying elements in the second phase. The EDS results show that the content of Mn in the MgZn_2_ in the ZTM441 alloy is 5.73 at.%. The measured lattice parameters of the MgZn_2_ are a = b = 5.985 Å, c = 9.284 Å, obviously larger than the theoretical values a = b = 5.223 Å and c = 8.566 Å, which may be caused by the solid solution of Mn element in the MgZn_2_ phase. Similarly, the content of Zn in the α-Mn in the ZTM441 alloy is 3.35 at.%. The measured lattice parameters are a = b = c = 10.108 Å, obviously larger than the theoretical values a = b = c = 8.913 Å, which may be caused by the solid solution of Zn element in the α-Mn phase.

In the alloys, the eutectic microstructure, Mg-Zn phase, and Mg-Sn phase will dissolve in the subsequent heat treatment, while α-Mn and Al_3_Mn_2_ will still exist in the matrix. These dispersoids can provide nucleation particles for dynamic recrystallization in the subsequent hot processing process, thus playing a role in refining grains and increasing the strength of the alloy [68,69,70]. The nanoscale Zn/Sn-rich phase in the gray-contrast zone is likely to precipitate during the slow cooling of cast ingot. Therefore, during the solid solution treatment process, re-dissolution will occur so as to obtain a high saturation solid solution, providing conditions for subsequent aging treatment. Two precipitation sequences can be achieved via subsequent appropriate aging processes, such as double-stage aging. The strength and toughness of the alloy can be improved by the MgZn_2_ and Mg_2_Sn precipitates formed along the prismic plane and base plane, respectively [71,72]. To sum up, via the study of the microstructure in the as-cast alloys, it can be found that a variety of phases can be formed in the alloy system, and via subsequent processing and heat treatment, multiphase composite strengthening and toughening can be achieved. The Mn element can also enhance the corrosion resistance of the alloy. Therefore, the alloy is expected to develop into a new magnesium alloy with excellent comprehensive properties.

## 5. Conclusions

In this study, the crystal structures, composition, and distribution characteristics of the intergranular second phases and intragranular dispersoids in ZTM441 and ZTM441-0.3Al as-cast alloys were investigated using FIB, SEM, EBSD, EDS, SAED, HRTEM, and HAADF-STEM. The main conclusions are as follows:

(1) In ZTM441 as-cast alloy, skeleton-shaped α-Mg+MgZn_2_ eutectic texture and block-shaped Mg_2_Sn formed along the grain boundary and the interdendrite. Nanoscale α-Mn dispersoids are distributed in the grain, and Zn/Sn-rich nanoscale precipitate bands are usually present along the grain boundary and the interdendrite. Part of the Zn element is dissolved into the α-Mn phase, while part of the Mn element is dissolved into the MgZn_2_ phase. Therefore, it can be concluded that Zn and Mn elements are easy to combine in ZTM441 as-cast alloy.

(2) In ZTM441-0.3Al as-cast alloy, skeleton-shaped α-Mg+Mg-Zn eutectic texture and block-shaped Mg_2_Sn formed along the grain boundary and the interdendrite. The Mg-Zn phases have not only a MgZn_2_-type crystal structure but also Mg_4_Zn_7_- and Mg_149_Zn-type crystal structure. Nanoscale Al_3_Mn_2_ particles were present in the grain, while no α-Mn formed. Zn/Sn-rich nanoscale precipitate bands are usually present along the grain boundary and the interdendrite. Zn is not dissolved into the α-Mn phase, and Mn is not dissolved into any Mg-Zn phases, which is different from the element distribution in ZTM441 as-cast alloy. Therefore, it can be concluded that the addition of Al changes the combination of Mn and Zn, while Al and Mn are easy to combine in ZTM441-0.3Al as-cast alloy.

(3) The average grain size of the ZTM441 as-cast alloy is 215.632 μm, and the average grain size of the ZTM441-0.3Al as-cast alloy is 398.797 μm. In ZTM441-0.3Al as-cast alloy, the addition of Al promoted the formation of Al_3_Mn_2_.

## Figures and Tables

**Table 1:** Actual compositions of the as-cast alloys in this work (wt.%).

Sample Type	Zn	Sn	Mn	Al	Mg
ZTM441	3.80	3.98	0.98	—	Bal.
ZTM441-0.3Al	4.00	4.06	0.86	0.29	Bal.

**Table 2:** Composition of the labeled second phases in ZTM441 as-cast alloy in Figure 3 (at.%).

	Mg	Zn	Sn	Mn	
A	96.19	1.84	1.54	0.44	Matrix
C	71.79	26.90	0.27	1.03	Zn-rich
D	15.75	0.00	0.00	84.25	Mn-rich
E	81.88	1.83	16.29	0.00	Sn-rich
F	64.49	6.40	29.12	0.00	Sn-rich
G	77.11	17.40	4.68	0.81	Zn-rich
H	80.81	18.45	0.74	0.00	Zn-rich

**Table 3:** Composition of the labeled second phases in ZTM441-0.3Al as-cast alloy in Figure 4 (at.%).

	Mg	Zn	Sn	Mn	Al	
C	7.67	0.00	0.00	61.97	30.35	Al-Mn-rich
D	81.65	17.21	0.49	0.14	0.51	Zn-rich
E	80.79	0.80	18.41	0.00	0.00	Sn-rich

**Table 4:** Composition of the labeled second phases in ZTM441 as-cast alloy in Figure 6a (at.%).

	Mg	Zn	Sn	Mn	
A	27.98	66.24	0.05	5.73	MgZn_2_
B	68.94	0.20	30.33	0.53	Mg_2_Sn
C	95.41	3.02	1.26	0.31	α-Mg

**Table 5:** Composition of the labeled second phases in ZTM441-0.3Al as-cast alloy in Figure 11a (at.%).

	Mg	Zn	Sn	Mn	Al	
A	59.49	0.25	39.97	0.11	0.18	Mg_2_Sn
B	20.84	78.81	0.07	0.16	0.12	MgZn_2_
C	37.69	61.39	0.05	0.08	0.79	Mg_149_Zn
D	29.34	69.67	0.08	0.12	0.80	Mg_4_Zn_7_
E	35.75	63.52	0.07	0.07	0.59	MgZn_2_

## Data Availability

The data presented in this study are available on request from the corresponding author.
